# ESCP best practice: development, implementation and evaluation of sick day guidance in primary care in the Netherlands

**DOI:** 10.1007/s11096-026-02097-0

**Published:** 2026-02-25

**Authors:** Tristan Coppes, Ellen S. Koster, Daphne Philbert, Teun van Gelder, Marcel L. Bouvy

**Affiliations:** 1 Department of Pharmacoepidemiology and Clinical Pharmacology, Faculty of Science, Utrecht Institute for Pharmaceutical Sciences (UIPS), Utrecht University, PO Box 80082, 3508 TB Utrecht, The Netherlands; 2 Education Center, University Medical Center Utrecht, Utrecht, The Netherlands; 3 Department of Clinical Pharmacy and Toxicology, Leiden University Medical Centre, Leiden, The Netherlands

**Keywords:** General practitioner, Implementation, Medication management, Pharmacy, Pharmacy technician, Primary care, Sick day guidance

## Abstract

**Introduction:**

To prevent acute kidney injury, guidelines recommend temporary adjustment of high-risk medications in patients with impaired renal function during so-called ‘sick days’. Sick days are periods with increased risk of dehydration, such as diarrhoea, fever or vomiting. Currently, awareness of sick day guidance among patients and healthcare professionals is low, which hampers implementation in daily practice.

**Aim:**

To develop, implement and evaluate sick day guidance for patients with pre-existing impaired renal function on maintenance treatment with high-risk medication.

**Setting:**

Over a 12 month study period, community pharmacies collaborated with at least one affiliated general practitioner (GP) to implement sick day guidance in primary care.

**Development:**

Training materials, including an E-learning module for healthcare professionals and patient information materials were developed.

**Implementation:**

In total, 21 community pharmacies completed the 12 month study period, in which 373 patients received oral and written instructions to report sick days to the GP or pharmacist. The median age of included patients was 78 years (IQR 73–83), 42% were male, and 68% used ≥ 2 high-risk medications. The implementation process of sick day guidance was evaluated with the Consolidated Framework for Implementation Research (CFIR), including a start interview in every pharmacy, registration of agreements, monthly telephone evaluations and an end-evaluation interview.

**Evaluation:**

Successful implementation was facilitated by adequate training, strong team engagement and support, and making pharmacy technicians responsible for information provision as well as providing in-person counselling to patients. Implementation barriers related to a lack of support from the information system, a lack of reimbursement and a low number of reported sick days. Patients reported 8 sick days, although a telephone survey amongst 188 patients showed that 12 more sick days had occurred.

**Conclusion:**

While training healthcare professionals supports appropriate medication adjustments when sick days are reported, patient education alone does not consistently lead to reporting. Additional implementation efforts, such as involving informal caregivers, may be needed to support patients in signalling and managing sick days.

**Supplementary Information:**

The online version contains supplementary material available at 10.1007/s11096-026-02097-0.

## Facilitators of best practice

- Close collaboration and task division for sick day management between the pharmacist and general practitioner.
- Training for the entire pharmacy and general practice team to ensure consistency in patient counselling and sick day recognition.
- In-person information provision to patients results in significantly better-informed patients.

## Barriers to best practice

- Complexity of sick day guidance information for some patients, which can be addressed by involving informal caregivers to support recognition and adherence.
- Hesitancy among pharmacists and general practitioners to temporarily adjust medication due to concerns about exacerbating underlying conditions, which can be overcome by clear agreements regarding patient monitoring during sick days.
- Limited involvement of home care organizations, which can be overcome by implementing sick day guidance region-wide across all relevant primary care professionals.

## Background

Approximately 10% of medication-related hospital admissions are caused by non-adjustment or wrongful adjustment of medications in patients with impaired renal function [1]. These patients are at an increased risk of developing acute kidney injury (AKI) [2, 3]. At the same time, many of these patients are on maintenance treatment with high-risk medications such as diuretics and renin–angiotensin system (RAS) inhibitors. While these drugs provide long-term benefits, they can precipitate AKI during episodes of dehydration [4]. Other commonly used drugs, such as SGLT2 (sodium glucose cotransporter 2) inhibitors, metformin and NSAIDs (non-steroidal anti-inflammatory drugs) (often referred to as SADMAN medications), can pose risks if they are used in periods where there is a risk of dehydration [5]. Such moments include external stressors like heatwaves and intercurrent diseases like vomiting, diarrhoea or high fever, often referred to as sick days.

During sick days, high-risk medications should be temporarily adjusted to prevent AKI. Research has shown that up to 40% of medication-related hospital admissions related to AKI or dehydration could potentially be prevented if medication is timely and adequately adjusted [6, 7]. Recently, national and international guidelines have provided more detailed information on sick day guidance [8–10]. And while sick day interventions have been studied previously [11, 12], application in daily practice and evaluation of these efforts remain limited [13]. An implementation study in the United Kingdom found there is a need for training of healthcare professionals and support of patients, and that roles and responsibilities need to be discussed to implement sick day guidance into routine practice successfully [14].

In the Netherlands, knowledge about the safe use of medication during sick days is low amongst high-risk patients with chronically impaired renal function [15–17]. Additionally, general practitioners (GPs), hospital-based physicians and pharmacists do not regularly apply sick day guidance in clinical practice [18]. Research in the Dutch setting suggests that the successful implementation of sick day guidance in clinical practice requires adequate training, effective collaboration among healthcare professionals, clearly defined roles and the provision of appropriate information materials for patients and healthcare professionals [19].

Community pharmacies can play a key role in implementing sick day guidance within primary care [19, 20]. They are easily accessible for patients seeking advice, including self-care-related questions, are well-informed about the patient’s medication use, and possess expertise regarding the effects of medication on renal function [21]. In addition, Dutch community pharmacists typically collaborate closely with GPs in their locality [22]. This positions them well to provide sick day guidance to patients, either at the point of dispensing or during medication reviews.

### Aim

This study aimed to develop, implement and evaluate sick day guidance for patients with pre-existing impaired renal function and on maintenance treatment with high-risk medication in primary care in the Netherlands.

## Development

### Design and stakeholder involvement

A needs assessment was conducted with relevant stakeholders, including patients, informal caregivers, pharmacists, GPs, nephrologists, and cardiologists. These findings have been published previously [16, 19]. In short, current knowledge and application of sick day guidance is low amongst patients and healthcare professionals in the Netherlands. There is a need for information materials for both patients and healthcare professionals, as well as a clear division of tasks and responsibilities between different healthcare professionals. Based on these findings, the necessary information materials and implementation strategies were designed.

### Intervention design and task division

**Fig. 1:**
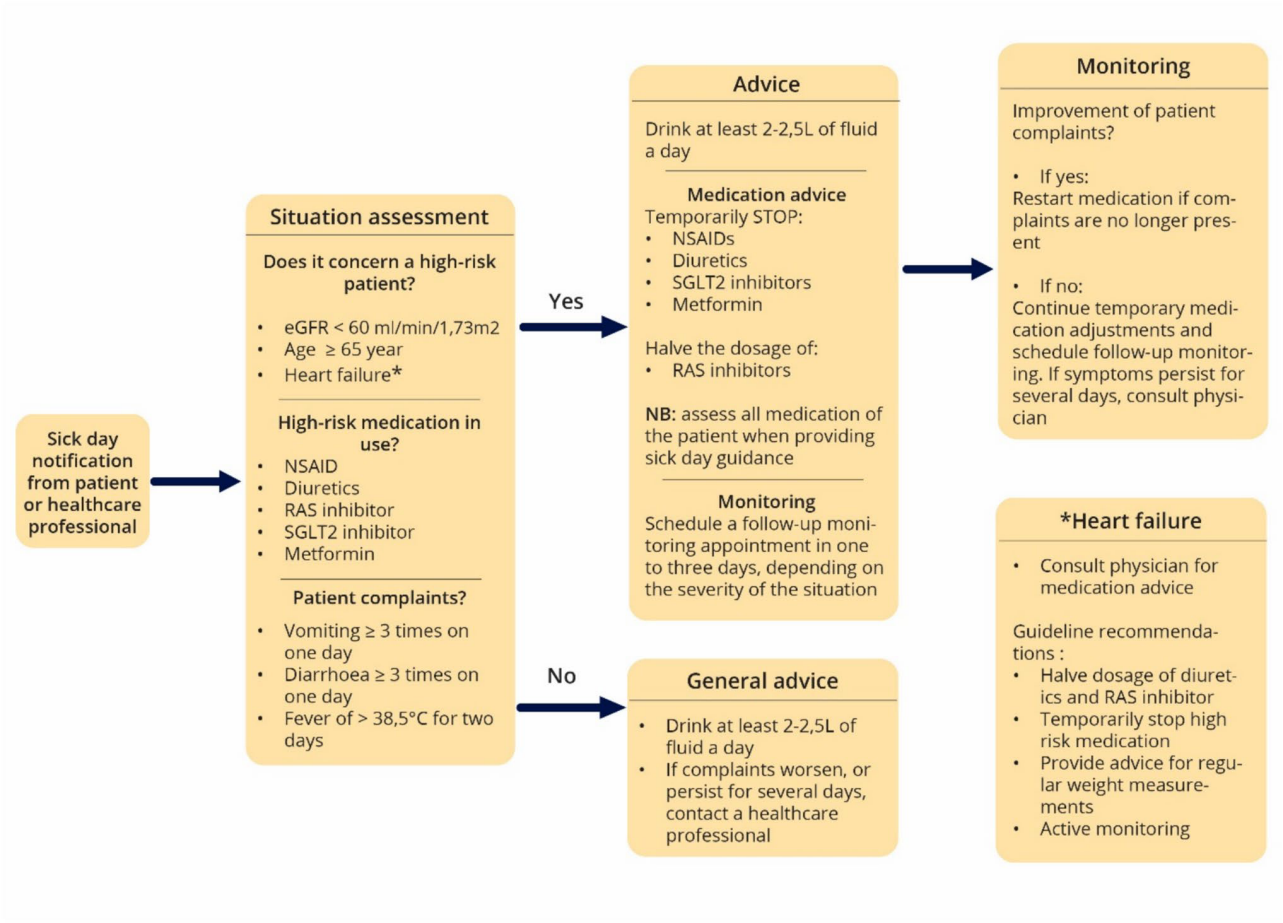
The default sick day guidance flowchart that was discussed during the initiation meeting and academic detailing session with pharmacist(s) and general practitioner(s)

Community pharmacies served as the coordinator for implementation in this study and were eligible for participation if at least one affiliated GP agreed to collaborate. During a scheduled initiation meeting with the pharmacist(s) and GP(s), the researchers conducted academic detailing and allocated responsibilities such as determining who would monitor patients during sick days, who patients should contact during sick days and who would be responsible for restarting medication after temporary adjustments. Additionally, participating pharmacists and GPs received a default sick day guidance flowchart based on the Dutch general practice guideline on chronic kidney disease [10] (Fig. 1), which they could adapt to their specific needs and work procedures, ensuring alignment with existing workflows. For example, they could decide whether patient inclusion would be carried out by pharmacists or pharmacy technicians, and whether patients should contact the pharmacy or the GP’s office in the event of a sick day.

### Training and materials for implementation

To support the different stakeholders during implementation, several training and information materials were developed. For pharmacy and GP staff, an e-learning module was developed to enable them to recognise high-risk patients and signal sick day complaints of patients. To support pharmacies in providing adequate information to the patient, a sick day guidance conversation protocol was developed. A patient information leaflet made by the Dutch Kidney Foundation was used, with information on sick days and how and when to temporarily adjust medication [23]. A smaller pocket-sized card was developed with instructions on when to contact a healthcare professional. These materials, including additional materials such as waiting room information slides, were available for healthcare professionals and patients on a website designed for this study (https://sidrik.sites.uu.nl/ (Dutch)).

## Implementation

### Pharmacy participation and patient recruitment

A total of 28 pharmacies attended the on-site initiation meeting, including an academic detailing session hosted by the researchers. Academic detailing entails a structured educational outreach program in which trained health professionals provide tailored evidence-based information to practicing healthcare professionals in their settings [24]. Following the academic detailing session, the researchers met with the pharmacist to address any remaining questions or concerns. Hereafter, pharmacists could start patient inclusion. Twenty-one pharmacies started patient inclusion and completed the 12-month follow-up. For more details on the included patients, see Table 1.

**Table 1:** Patient characteristics of all included patients

Variable	Number of patients (N = 373)
Sex, female, n (%)	174 (47)
Age in years, median (IQR)	78 (73–83)
Estimated glomerular filtration rate in mL/min/1.73 m^2^, median (IQR)	45 (38–52)
Medication in use:	
Diuretic, n (%)	315 (84)
ACE-inhibitor, n (%)	234 (63)
ARB, n (%)	95 (25)
Metformin, n (%)	110 (29)
SGLT2-inhibitor, n (%)	57 (15)
NSAID, n (%)	5 (1)
Two or more high-risk medication in use, n (%)	320 (68)
Three or more high-risk medication in use, n (%)	127 (34)
Living at home, n (%)	365 (98)
Individualized dispensing system in use, n (%)	102 (27)

*IQR* Interquartile range, *ACE-inhibitor* angiotensin converting enzyme inhibitor, *ARB* angiotensin receptor blocker, *SGLT2-inhibitor* sodium glucose cotransporter 2 inhibitor, *NSAID* non-steroidal anti-inflammatory drug

Twelve of the 21 pharmacies were situated in a health centre together with the GP. Two pharmacies had fewer than 5000 registered patients, seventeen pharmacies had between 5000 and 15,000 registered patients, and two pharmacies had more than 15,000 registered patients.

Patients were eligible if they had impaired renal function (eGFR below 60 mL/min/1.73 m^2^), were older than 65 years, and used at least one high-risk SADMAN medication. Patients were recruited by the pharmacy during a counselling session, conducted either by telephone or in person. In the Netherlands, the renal status of a patient is, in most cases, available to the community pharmacists as it is mandatory to share this information in the primary care chain [25]. All patients received an information leaflet and a pocket card. Throughout the 12 month follow-up period, pharmacies documented every sick day report from included patients using a standardised data collection form, see Supplementary material S1. This form recorded details on medication adjustments made, as well as the agreed-upon monitoring.

### Local adaptation of implementation strategies

Eleven settings chose to inform the patient to report a sick day to the pharmacy, two settings chose to have sick days go through the GP office, and eight settings decided to instruct the patient to report a sick day to either one of them. Main differences in implementation strategies are shown in Table 2. Implementation strategies could also change during implementation if it was thought to be beneficial for implementation.

**Table 2:** Overview of different implementation strategies of pharmacies

	Number of pharmacies (n = 21)
Information primarily provided by	
Pharmacist, n (%)	11
Pharmacy technician, n (%)	6
Pharmacist in training, n (%)	4
Information provided through	
Telephone, n (%)	9
In person, n (%)	12
Patient is instructed to report sick days to	
Pharmacy, n (%)	11
General practice, n (%)	2
Pharmacist or general practice, n (%)	8

### Ethics approval

This study was conducted in compliance with the requirements of the Institutional Review Board of the Division Pharmacoepidemiology and Clinical Pharmacology, Utrecht (reference number: UPF2216, date: 03-02-2023). All participating pharmacies provided written informed consent before study initiation. Additionally, all patients had to sign an informed consent form to participate. Patients could deny participation in this study but still receive sick day guidance information.

### Evaluation methods

Both qualitative and quantitative data were collected to evaluate the implementation process. The quantitative data consisted of inclusion numbers, sick day reports and level of knowledge and number of sick days obtained through the telephone evaluations with patients. The qualitative data were collected through the registration and agreement form, interviews held at the start (this included a context analysis interview to understand the organisational and environmental context in which implementation took place), during (monthly telephone evaluations) and at the end of implementation (evaluation interview) with pharmacists, see Supplementary material S2 for an overview of all evaluation methods. All interview guides were developed and analysed using the Consolidated Framework for Implementation Research (CFIR), see Supplementary material S3 for the start- and end-evaluation interview guide. Data analysis was performed using NVIVO version 14 coding software; see Supplementary material S4 for the coding tree.

### Patient inclusion and sick day reports

A total of 373 high-risk patients received verbal and written information on sick day guidance in a counselling session. The median age of patients was 78 years (IQR: 73–83). Sixty-eight percent of the included patients used two or more high-risk medications, and 34% used three or more high-risk medications. A total of 8 sick day reports in 373 patients (2.1%), divided over four pharmacies, were registered in this study. During telephone evaluations with 188 (50%) patients, it was found that 12 patients had experienced sick days but had not contacted any healthcare professional or made temporary medication adjustments despite previous attendance of the counselling session. There was a statistically significant difference in knowledge level between patients who received oral counselling in person (either at the pharmacy or at home) and those who received counselling via telephone, χ^2^ = 48.11, p < 0.001, see Table 3. For this analysis, the knowledge level was separated into no mention of high-risk moments, compared to mentioning at least one or more high-risk moment.

**Table 3:** Knowledge level of patients regarding high-risk sick day moments in patients who received in-person counselling vs. telephone counselling

Knowledge level (%)	Telephone counselling (n = 55)	In-person counselling (n = 133)
No recollection of the counselling moment, n	20 (36.4)	9 (6.8)
Remembers the counselling moment, but content is unknown, n	30 (54.5)	38 (28.6)
Remembers 1 of 4 high-risk moments*, n	3 (5.5)	40 (30.1)
Remembers 2 of the high-risk moments, n	0 (0.0)	19 (14.3)
Remembers 3 of the high-risk moments, n	1 (1.8)	19 (14.3)
Remembers all and knows how to act, n	1 (1.8)	8 (6.0)

*High-risk moments include: heatwaves, vomiting, diarrhoea, and high fever

## Evaluation

### Perceived value of sick day guidance

At the interviews at the start of the study, pharmacists highlighted sick day guidance as an important way to reduce preventable hospital admissions, increase pharmacy team knowledge and capabilities, and demonstrate the pharmacy’s value to patients. At the evaluation interviews after implementation, however, reducing hospital admissions was no longer mentioned, as this outcome was not measurable during the study due to the limited number of sick day reports. This demotivated a few participants, who stated that participation ultimately did not feel worth the effort.

“If you look at the hours we put into this project, we don’t think it has been directly worth the effort […], we could have put these hours into another topic which could have provided more direct results” (Pharmacist 9)

However, after implementation, all pharmacists remained positive about the value of increased pharmacy team knowledge and awareness about sick days.

### Compatibility of the innovation

A key obstacle in implementation was integrating sick day guidance into daily practice. Some pharmacies planned separate counselling sessions with high-risk patients, but this required extra planning outside of existing workflows. Other pharmacies incorporated sick day counselling in medication reviews, yet most pharmacies abandoned this approach due to the high time investment and the tendency for sick day guidance to be overshadowed by other topics during medication review.

“My original plan was to incorporate the information provision in a medication review, however, I noticed that this took a lot of time, and patients come up with all kinds of different topics, which made it difficult to stay focused on sick day management. So later on, I discontinued incorporating the information in medication reviews” (pharmacist 19)

### Challenges in sustaining stakeholder engagement

Keeping stakeholders such as GPs and home nursing organisations engaged proved challenging, largely due to the low number of sick day reports from patients. As a result, the involved healthcare professionals gradually lost sight of the agreements and workflows concerning sick day guidance that had previously been designed.

“Yes, the agreements were clear, and everyone knew what to do at the time. But if the situation barely occurs afterwards and months later, someone might call, and you think, ‘Wait, what was that again?’ Then there’s no routine, and those agreements kind of get lost.” (pharmacist 8)

Pharmacists also mentioned difficulties involving home nursing organisations, mainly because the presence of multiple organisations in the same area made communication and a harmonised workflow hard to implement.

“We don’t really work much with home nursing services. Of course, some of our patients receive care at home, [..] but in our area there are just so many different ones, and that makes it quite complicated" (pharmacist 2)

### Needs and resources of patients

During implementation, many pharmacies reported patient-related barriers to sick day guidance, as patients often reacted that they were never sick and thought that sick day guidance did not apply to them. Additionally, sometimes the information appeared too difficult for patients to understand, or patients preferred the GP to be in charge of medication changes instead of the pharmacist.

“I got the feeling that patients downplay the seriousness of the information and that they think it will not happen to them personally. They often say: Oh, but I am never sick, this does not happen to me”. (pharmacist 6)

### Conditions for the sustainment of sick day guidance efforts

A frequently mentioned requirement for sustainment concerned software capabilities. Currently, most pharmacy information systems lack alerts or prompts about sick day guidance for high-risk patients, forcing pharmacists and pharmacy technicians to manually identify eligible patients, which takes extra time and effort and hinders implementation.

“We need our information system to provide better signals. For example, if the patient has impaired renal function, a signal should pop up when a high-risk medicine is prescribed with an instruction to provide information about sick day guidance” (pharmacist 3)

Due to the significant time investment and the extra service of pharmacies providing sick day guidance, remuneration was mentioned as another important factor for future sustainability.

“My healthcare heart tells me that this [providing sick day guidance information] is important to do, and that’s why we participated. However, if I were critical and wanted to continue, we should get compensated because we are providing an extra service” (pharmacist 17)

Pharmacies that underperformed and did not meet inclusion targets, or were dissatisfied, mentioned unforeseen staff shortages or simultaneous impactful changes in the pharmacy, e.g. a switch of pharmacy information system or a renovation as important implementation and sustainment barriers.

### Best practices in sick day guidance implementation

The seven pharmacies with the highest patient inclusion numbers (24–69 patients) accounted for seven of the eight collected sick day reports. Importantly, all sick day reports were collected from pharmacies that provided direct, in-person counselling, either in the pharmacy or during home visits. These high-performing pharmacies, which reported greater satisfaction and more successful implementation of sick day guidance, identified several key facilitators for implementation. First, involving and educating the entire pharmacy team allowed pharmacy technicians to take responsibility for sick day guidance counselling to patients and enabled them to better recognise sick days from patients. Secondly, scheduled in-person patient counselling facilitated patient understanding, allowed for the resolution of questions, and enabled the participation of partners or informal caregivers in the counselling session. Thirdly, close collaboration with the GP office and other healthcare professionals helped to harmonise workflows and patient communication.

”I think the information is more likely to be remembered when provided in person compared to a phone call, because patients get calls from the pharmacy all the time. A phone message makes less of an impression than sitting down with them in the consultation room and showing the information physically, for example, using the pocket cards.” (pharmacist 1)

“I’m good at initiating the process, but then someone else has to take over to continue it. You also need someone for that handover, so including everyone and continuity within the pharmacy team is essential.” (pharmacist 12)

Lastly, among the high-performing pharmacies, three pharmacies changed their counselling session during implementation to better fit the needs of patients and the network around the patient. After some inclusions, they started to also invite the partner or informal caregiver of the patient to the sick day counselling session to improve information retention and ensure that someone other than the patient could take action when needed.

“For the first counselling sessions, we weren’t very attentive to whether caregivers or family members accompanied the patient. Later on, we encouraged patients to bring their partner or informal caregiver so that two people would hear the information. That way, it’s more likely to stick, since the caregiver is often the one who needs to act because the person who’s ill usually doesn’t reach out themselves.” (pharmacist 16)

## Discussion

Although pharmacists and GPs collaborated in implementing sick day guidance, integrating it into daily practice remains challenging. A single counselling session with patients results in a limited number of sick day reports. Based on previous research, key needs and requirements of both healthcare professionals and patients, such as clear medication flowcharts, a well-defined division of tasks and responsibilities between pharmacists and GPs, patient information materials, and training for healthcare professionals, were incorporated into the study design. Despite addressing these factors, successful implementation remained challenging.

Previous research shows a clear need for accessible and understandable information materials to support patients during sick days [16]. A key barrier found in this study was misalignment between the provided information and instructions and the actual needs, preferences and health literacy of the patients. As found in previous literature, a limited health literacy is common in patients with impaired renal function influencing adherence to application of instructions [13, 26]. Our previous needs assessment also revealed that informal caregivers often showed a greater interest in receiving and understanding sick day information than the patients themselves. Although pharmacies were encouraged to involve informal caregivers or partners in the information provision, only a few implemented this practice during the study, making it difficult to evaluate its effectiveness. Nevertheless, pharmacies that adopted this approach reported positive experiences, noting that it improved knowledge retention and ensured that someone could act on the patient’s behalf when they were unwell. Caregivers were seen as valuable in recognising symptoms and contacting the pharmacist or GP for the patient. However, ethical and legal considerations regarding patient confidentiality and privacy may limit caregiver involvement, even when it could support patient care. Clear institutional guidelines and training are needed to help professionals balance data protection with the benefits of caregiver engagement.

Several pharmacists in this study reported that patients often indicated the information did not apply to them, frequently noting that they rarely experience illness and feel healthy, and therefore did not perceive the guidance as relevant. This demotivated some pharmacists from continuing with the information provision of sick day guidance. This is in contrast with previous research in New Zealand, where patients demonstrated willingness to self-manage sick days [27]. This can be explained by the slightly higher age of the included patients in this study, because older patients might in general be more reluctant to engage with health-related information. Future implementation efforts should more actively engage the patient’s care network, including informal caregivers and home care nurses. In this study, including home nursing organisations proved to be challenging for the participating pharmacies due to the large number of organisations usually present in the area. This fragmentation hinders communication and consistent workflows.

In this study, it was challenging to prospectively estimate the number of sick days for the included patient group in one year. Previous research among adults suggests that possibly 5–10% of the population experiences sick day symptoms such as fever or diarrhoea at least once a year [11, 28–30]. For this study, however, only eight direct sick day reports from patients were registered during follow-up. The identified sick days were exclusively reported in pharmacies that conducted a scheduled, in-person counselling session with the patient, suggesting that more passive strategies, such as providing information through telephone or sending written materials, may be insufficient to enable patient action, as also found in the literature [31, 32]. Nevertheless, due to the limited number of observed sick days, further research is required to confirm these findings and assess the effectiveness of different information delivery methods. However, based on the results of this study, in-person information provision seems to be significantly better compared to telephone counselling for knowledge retention.

Forgetfulness and limited adherence of patients to the provided advice could be important reasons for the limited number of sick days reported [33, 34]. In this study, we intentionally included patients with pre-existing impaired renal function, as this group is at the highest risk of experiencing sick days. However, cognitive and functional vulnerabilities in this population may limit their capacity to understand and retain new health-related information [35]. This was also found in a previous sick day guidance implementation study in the United Kingdom, where the concept of temporary adjustment of medication proved to be challenging for populations with a high risk of acute kidney injury [14]. These considerations suggest that education on sick day management should ideally be introduced earlier in the treatment trajectory, such as at the initiation of SADMAN medications, so that the concept becomes familiar by the time patients should apply the information.

Additionally, there is an existing perception among patients that providing medical advice when unwell is primarily the role of GPs, rather than pharmacists [36, 37]. As such, this presents a barrier to pharmacy-led sick day guidance. Although this study aimed to shift certain responsibilities, such as medication-related advice, from GPs to pharmacists to reduce pressure on primary care, patients may not recognise pharmacists as a first point of contact in such situations.

A strength of this study was the longitudinal design with multiple data points, including a structured contextual analysis of participants, followed by monthly contact moments and an elaborate end evaluation. This allowed insights into expected and experienced barriers and facilitators in the complete process of implementation. Another strength was the use of the CFIR framework throughout the design of the data collection methods and analysis of the data, allowing interpretation and linking of information to different implementation domains, and thus providing a complete picture of influencing factors.

A limitation of this study is that GPs were less involved in the evaluation of the project compared to pharmacists. Pharmacists led the implementation throughout the study, and no formal evaluation or follow-up was conducted with GPs after the initiation meeting and the e-learning module. As a result, relevant perspectives from GPs may have been missed.

## Conclusion

This study identified key factors influencing the implementation of sick day guidance in primary care. Training pharmacy technicians and in-person counselling of patients improved patient counselling and recognition of sick days, while mismatches between information provided to patients and their needs, understanding and expectations were important barriers. Future sick day guidance implementation efforts should involve informal caregivers and home nursing organisations to strengthen the patient’s support network in signalling sick days to pharmacists or general practitioners.

## Supplementary Information

Below is the link to the electronic supplementary material.

Supplementary file1 (DOCX 19 kb)

Supplementary file2 (DOCX 15 kb)

Supplementary file3 (DOCX 19 kb)

Supplementary file4 (DOCX 842 kb)

## Data Availability

The datasets generated during this study are available from the corresponding author on reasonable request.
