# Supplementary material for: ESCP best practice: development, implementation and evaluation of sick day guidance in primary care in the Netherlands
Source: Int J Clin Pharm. 2026 Feb 25;48(2):667–76. doi: 10.1007/s11096-026-02097-0 (PMC12992379; doi:10.1007/s11096-026-02097-0)
Supplement: Supplementary file 1 — Supplementary file1 (DOCX 19 kb) [file 11096_2026_2097_MOESM1_ESM.docx]

| Patient research number:  Or:  Name:  Date of birth: | | Date of report: | Reported by:  Patient / informal caregiver / home care nurse / other |
| --- | --- | --- | --- |
| **Sick day notification** | Onset of patients symptoms: | | |
|  | Symptoms: Diarrhoea / vomiting / fever / reduced intake  additional information: | | |
|  | Advice given to patient: | | |
|  | Temporary medication changes: | | |
|  | Patient monitoring appointment:  After how many days:  How: | | |
|  |  | | |
| **Monitoring** | Has there been further contact with the patient?  By phone / in person | | |
|  | Patient’s condition at monitoring: | | |
|  | Medication restarted: Yes / No | | |
|  | Total duration of medication adjustment: | | |
|  | Form completed by: | | |
