# Supplementary material for: ESCP best practice: development, implementation and evaluation of sick day guidance in primary care in the Netherlands
Source: Int J Clin Pharm. 2026 Feb 25;48(2):667–76. doi: 10.1007/s11096-026-02097-0 (PMC12992379; doi:10.1007/s11096-026-02097-0)
Supplement: Supplementary file 2 — Supplementary file2 (DOCX 15 kb) [file 11096_2026_2097_MOESM2_ESM.docx]

**Supplementary table S2**

**Implementation of sick day guidance in primary care in the Netherlands**

**Authors:**

1. Tristan Coppes ^1^, ORCID: 0000-0003-2817-139X
2. Ellen S. Koster^2^
3. Daphne Philbert^1^,
4. Teun van Gelder^3^, ORCID: 0000-0001-5980-6947
5. Marcel L. Bouvy^1^,ORCID: 0000-0002-4596-0684

1. Department of Pharmacoepidemiology and Clinical Pharmacology, Utrecht Institute for Pharmaceutical Sciences (UIPS), Faculty of Science, Utrecht University, Utrecht, The Netherlands
2.Education Center, University Medical Center Utrecht, Utrecht, The Netherlands
3. Department of Clinical Pharmacy & Toxicology, Leiden University Medical Centre, Leiden, The Netherlands

**Corresponding author:** Marcel Bouvy, PO Box 80082, 3508 TB Utrecht, The Netherlands, m.l.bouvy@uu.nl, +31 (0)623013551

*Table S2: Overview of the quantitative and qualitative data collection efforts used for evaluation*

|  | Participants | Data collection |
| --- | --- | --- |
| Start of implementation |  |  |
| Site initiation meeting | Pharmacist and general practitioners | Structured collaboration form  and sick day guidance flowchart |
| Context analysis interview | Pharmacist | On-site semi-structured interview |
| During implementation |  |  |
| Monthly progress meeting | Pharmacist | Semi-structured telephone interview |
| Sick day check-up | Patients | Structured telephone interview |
| End of implementation |  |  |
| End evaluation interview | Pharmacist | On-site semi-structured interview |
| Final patient inclusion file | Pharmacist | Quantitative data file |
| Sick day report forms | Pharmacist and GP | Structured form |
