# Supplementary material for: ESCP best practice: development, implementation and evaluation of sick day guidance in primary care in the Netherlands
Source: Int J Clin Pharm. 2026 Feb 25;48(2):667–76. doi: 10.1007/s11096-026-02097-0 (PMC12992379; doi:10.1007/s11096-026-02097-0)
Supplement: Supplementary file 3 — Supplementary file3 (DOCX 19 kb) [file 11096_2026_2097_MOESM3_ESM.docx]

**Supplementary material S3: semi-structured interview guides used at the start and end of implementation**

**Implementation of sick day guidance in primary care in the Netherlands**

**Authors:**

1. Tristan Coppes ^1^, ORCID: 0000-0003-2817-139X
2. Ellen S. Koster^2^
3. Daphne Philbert^1^,
4. Teun van Gelder^3^, ORCID: 0000-0001-5980-6947
5. Marcel L. Bouvy^1^,ORCID: 0000-0002-4596-0684

1. Department of Pharmacoepidemiology and Clinical Pharmacology, Utrecht Institute for Pharmaceutical Sciences (UIPS), Faculty of Science, Utrecht University, Utrecht, The Netherlands
2.Education Center, University Medical Center Utrecht, Utrecht, The Netherlands
3. Department of Clinical Pharmacy & Toxicology, Leiden University Medical Centre, Leiden, The Netherlands

**Corresponding author:** Marcel Bouvy, PO Box 80082, 3508 TB Utrecht, The Netherlands, m.l.bouvy@uu.nl, +31 (0)623013551

**Interview guide start context analysis**

[Introduction]

**1. Characteristics of the Setting**

**Characteristics within the Pharmacy**

1. How would you describe the culture in your pharmacy?
   *(Think about beliefs regarding patient care, the general atmosphere at work, norms, and values.)*
2. Do you think this project aligns with your organization’s culture?
   a. How do you think the culture might influence the project?
3. Do you already have any agreements or protocols regarding the safe use of medicines in patients with reduced kidney function?
   - If yes, what do these agreements entail?
4. Do you have experience with previous projects in your pharmacy?
   - If yes, what kind of project was that?

**Characteristics Outside the Pharmacy**

1. Which other healthcare providers are actively involved in this project?
2. How would you describe the relationship with the other healthcare providers participating in the project?
   *(e.g., general practitioners, home care nurses)*
3. To what extent do you collaborate in a network with other healthcare providers outside your own setting?
4. What kind of information exchange takes place between you and them? Both regarding work processes and general communication?

**2. Implementation of Sick Day Guidance**

**Implementation Process**

1. In your opinion, what is the added value of implementing *sick day guidance*?
2. How complex do you find the procedure?
   *(Consider factors such as duration, patient reach, number of steps, and the degree of adjustment to existing care routines.)*
3. What do you see as the biggest obstacle to implementing the procedure?
   a. What other challenges do you anticipate?
4. What might help you in carrying out the project?
5. Can you integrate sick day guidance into existing workflows?
   - How do you plan to do that?
     *(e.g., during polypharmacy consultations or when patients collect their medication at the counter)*
6. Who from the pharmacy team will be involved in the project?
   - *(e.g., pharmacy assistants, consultants)*
     a. What responsibilities will they have?

**Available Resources**

1. Do you feel that you have sufficient support from the research team to implement the procedure effectively (in terms of guidance and materials)?
2. What additional materials or support would you like to receive?

**3. Beliefs and Goals**

**Process-Related Topics**

1. What concrete goals would you like to set for this project?
2. To what extent do you monitor whether these goals are being achieved? How do you plan to do that?
3. How will you ensure that all pharmacy staff are engaged and supportive of the project?
   *(e.g., through internal staff meetings)*
   a. What obstacles do you expect?
4. Do you have a timeline or plan for rolling out the project?
5. How will the e-learning module be shared, and will it be mandatory for staff to complete?
6. How will you ensure that healthcare providers outside the pharmacy are also supportive of the project?
   a. What obstacles do you expect?

**Personal Beliefs about the Project**

1. Do you think the project will be effective?
   - In other words, do you expect to receive *sick day* notifications from patients? Why or why not?
2. How confident are you that the project can be implemented as intended in your setting? Why?

**4. Beliefs about Patients**

**Patient Needs and Preferences**

1. What barriers do you expect patients might face when participating in the project?
2. What could be done to reduce or remove these barriers?
3. How capable do you think patients are of recognizing a *sick day* themselves?

**Interview guide end evaluation interview**

[Introduction]

**Project Execution**

1. Who were the main implementers of the project within your pharmacy?
   a. Is everyone in the team aware of the project and its objectives?
   b. Did you make use of the e-learning module?
2. How was the collaboration with other healthcare professionals?
   *(e.g., general practitioners, home care providers, secondary care professionals)*
   a. Are you satisfied with this collaboration?
   b. How would you ideally like this collaboration to look?
3. Were you able to reach the patients you intended to target with the project?
   a. Why or why not?
   b. Were caregivers (informal carers) involved?
4. Are there certain patient groups that you were less able to reach?
   a. Why or why not?
5. If you could start over, would you use different patient selection criteria?

**Informing Patients**

1. How did the process of informing patients go in your pharmacy?
   a. To what extent were you able to implement patient education as initially planned?
   *(for example, using the counselling agreement form)*
   b. During the interim evaluation, several adjustments were proposed. How were these carried out?
   c. Did these adjustments lead to the intended effect?
2. In your opinion, was informing patients worthwhile for this patient group?
   a. Why or why not?
   b. How would you ideally like to implement patient education in daily practice?
   c. Do you think pharmacy assistants could play a role in this process?
   d. Would you be more inclined to do this if financial compensation were provided?
3. What has been the time investment for this project?
   a. On average, how much time does it take to inform a single patient?

**Sick Day Reports**

1. In your opinion, has the implementation of the *sick day guidance* been effective?
   a. Why or why not?
2. What do you think is the main reason for the relatively low number of *sick day* reports from patients?
   a. Do you think patients are motivated to act on the advice provided?
   b. Do you think patients understood the advice and knew what was expected of them?
   c. Is it possible that some *sick day* notifications were not recognized as such by pharmacy or GP assistants?
3. What changes would need to be made to the current strategy in order to increase the number of *sick day* reports?
4. Have there been any unintended outcomes resulting from informing patients?
   a. If yes, could you elaborate?

**Implementation**

1. What do you consider to be key successful elements of the implementation process?
   *(For example: specific activities, materials, roles, or qualities of certain individuals.)*
2. What were the main challenges or obstacles encountered during implementation?
3. What characteristics of your pharmacy facilitated the implementation process?
   a. What made it possible for implementation to succeed in your setting?
   b. The project has been implemented in many pharmacies, each adapting it to their own context. What were the success factors in your pharmacy?
   c. Are there aspects that are unique to your pharmacy that contributed positively to implementation?
4. What characteristics of your pharmacy or setting made implementation more difficult?
5. To what extent has the procedure become part of daily routine activities?
6. What plans are in place to sustain the project within your setting?
7. Are there plans to scale up the procedure to other settings?
   a. What specific plans exist for this?
   b. What barriers do you expect?
   c. What kind of support would you need for this, potentially from our research team?

**Closing Questions**

1. What has participation in this project brought to your pharmacy?
2. What would you do differently if you were to carry out the project again?
3. From your perspective, what should be the next step for the research team to further support practice?
4. Are there any topics that have not been discussed that you would still like to share?
