# Supplementary material for: ESCP best practice: development, implementation and evaluation of sick day guidance in primary care in the Netherlands
Source: Int J Clin Pharm. 2026 Feb 25;48(2):667–76. doi: 10.1007/s11096-026-02097-0 (PMC12992379; doi:10.1007/s11096-026-02097-0)

**Supplementary material S4: Codetree**

**Implementation of sick day guidance in primary care in the Netherlands**

**Authors:**

1. Tristan Coppes ^1^, ORCID: 0000-0003-2817-139X
2. Ellen S. Koster^2^
3. Daphne Philbert^1^,
4. Teun van Gelder^3^, ORCID: 0000-0001-5980-6947
5. Marcel L. Bouvy^1^,ORCID: 0000-0002-4596-0684

1. Department of Pharmacoepidemiology and Clinical Pharmacology, Utrecht Institute for Pharmaceutical Sciences (UIPS), Faculty of Science, Utrecht University, Utrecht, The Netherlands
2.Education Center, University Medical Center Utrecht, Utrecht, The Netherlands
3. Department of Clinical Pharmacy & Toxicology, Leiden University Medical Centre, Leiden, The Netherlands

**Corresponding author:** Marcel Bouvy, PO Box 80082, 3508 TB Utrecht, The Netherlands, m.l.bouvy@uu.nl, +31 (0)623013551


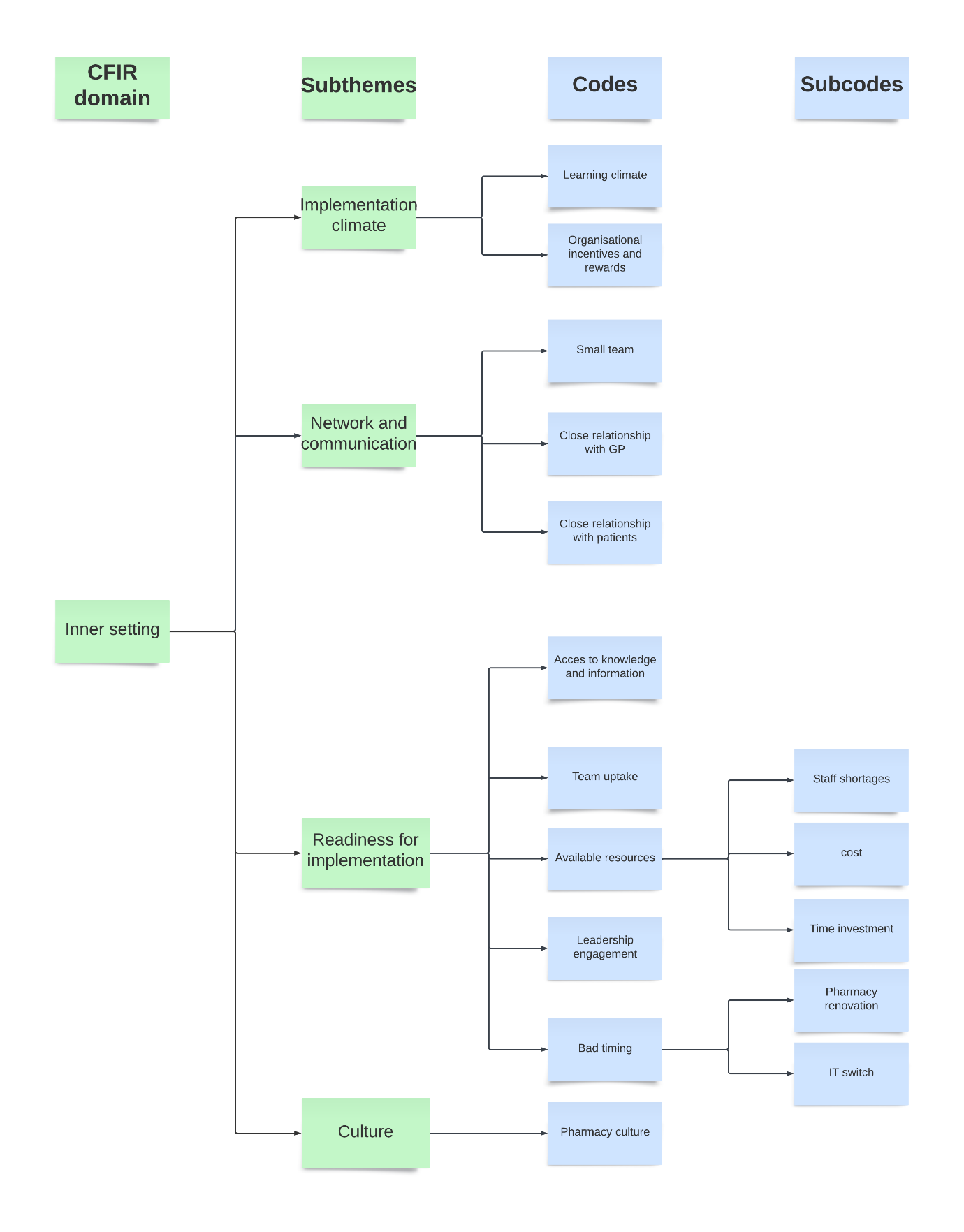


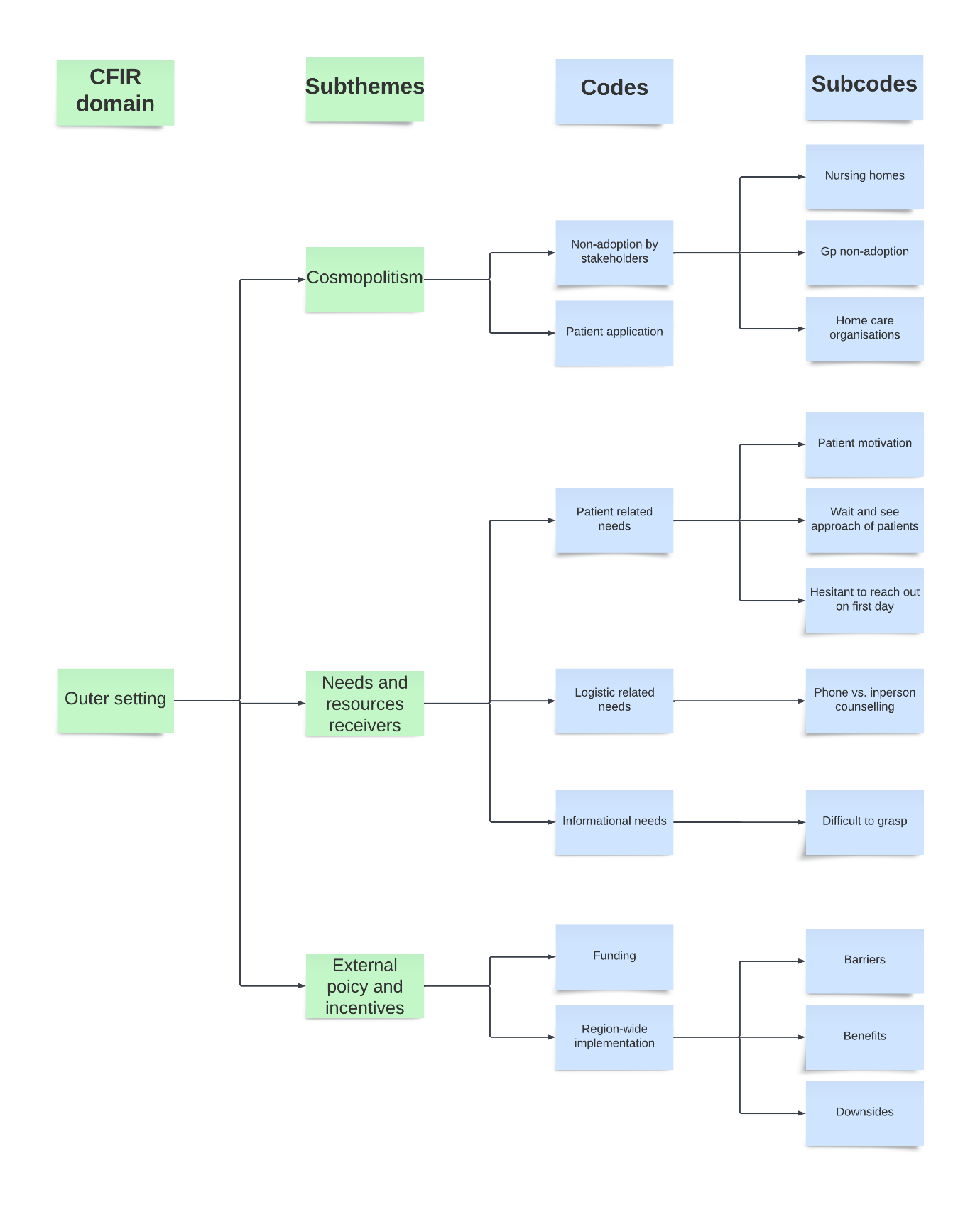


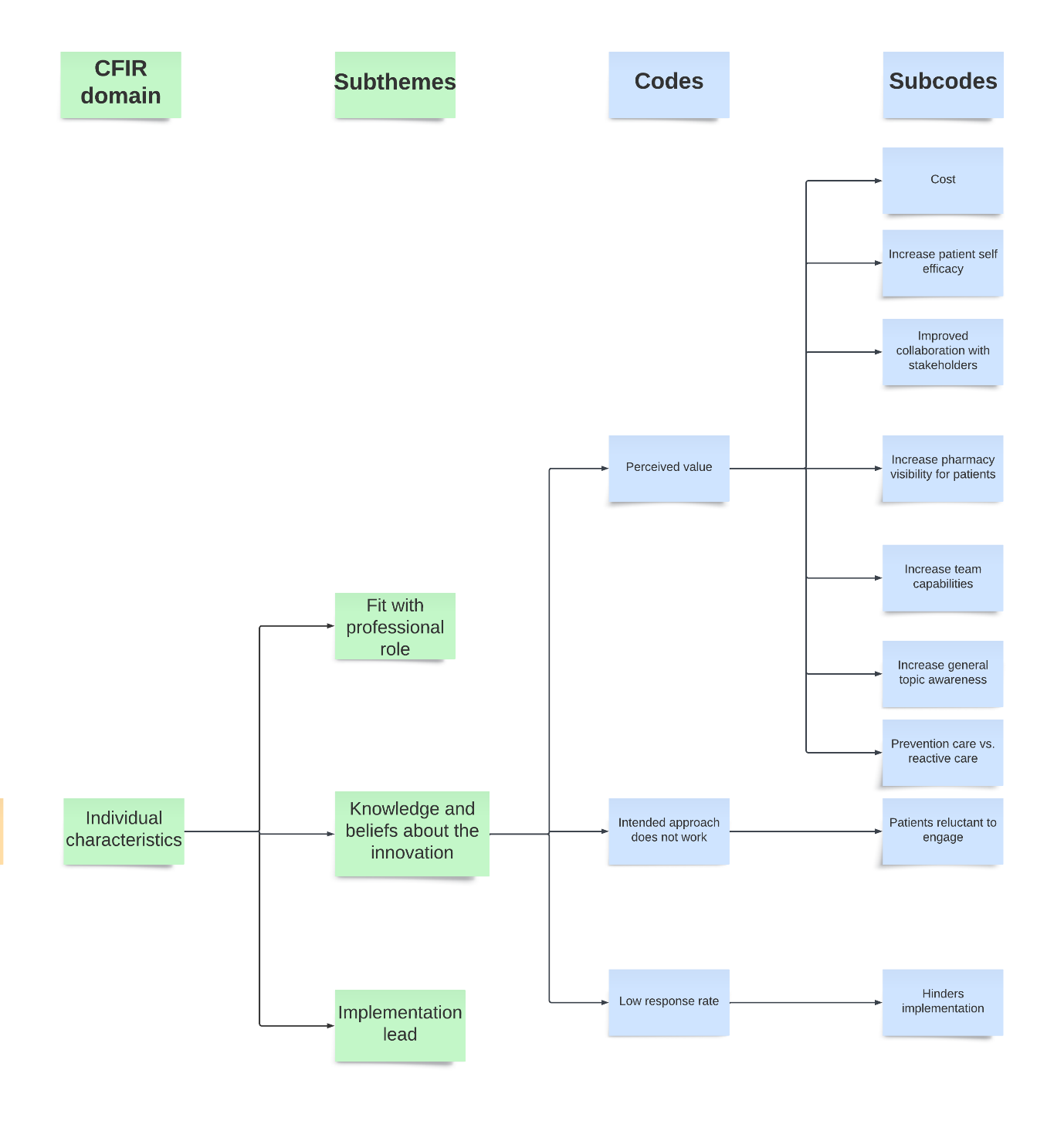


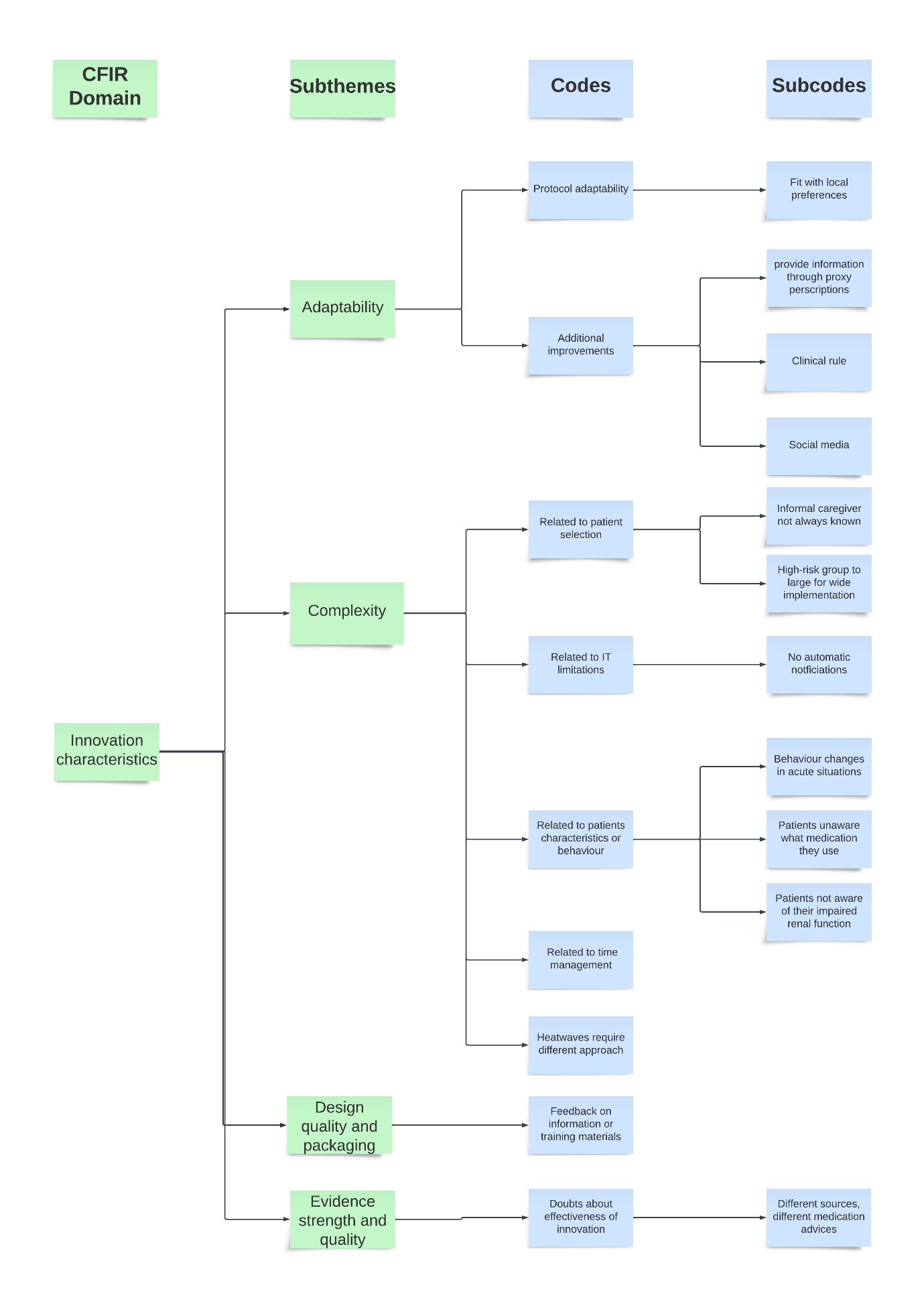


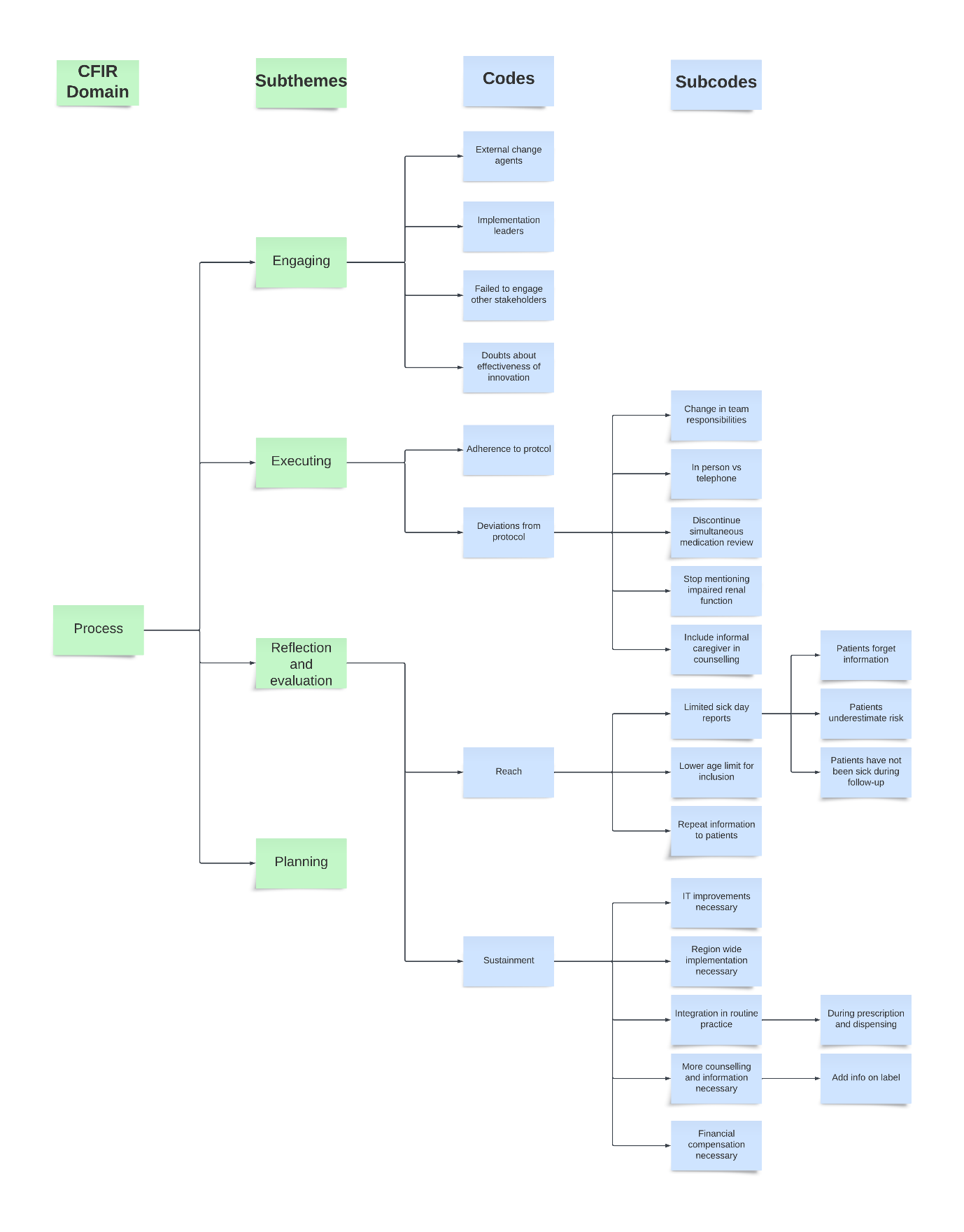

Supplement: Supplementary file 4 — Supplementary file4 (DOCX 842 kb) [file 11096_2026_2097_MOESM4_ESM.docx]
